# *Staphylococcus aureus* Proteins Implicated in the Reduced Virulence of *sarA* and *sarA/agr* Mutants in Osteomyelitis

**DOI:** 10.3390/microorganisms13010181

**Published:** 2025-01-16

**Authors:** Karen E. Beenken, Mara J. Campbell, Stephanie D. Byrum, Rick D. Edmondson, Samuel G. Mackintosh, Alan J. Tackett, Mark S. Smeltzer

**Affiliations:** 1 Department of Microbiology and Immunology, University of Arkansas for Medical Sciences, Little Rock, AR 72205, USA; mjcampbell@uams.edu (M.J.C.); smeltzermarks@uams.edu (M.S.S.); 2 Department of Biochemistry and Molecular Biology, University of Arkansas for Medical Sciences, Little Rock, AR 72205, USArdedmondson@uams.edu (R.D.E.); mackintoshsamuelg@uams.edu (S.G.M.); ajtackett@uams.edu (A.J.T.)

**Keywords:** *Staphylococcus*, osteomyelitis, *sarA*, *agr*, protease, proteomics

## Abstract

Using a murine osteomyelitis model, we recently demonstrated that *Staphylococcus aureus sarA* and *sarA/agr* mutants generated in the USA300 strain LAC are attenuated to a greater extent than an isogenic *agr* mutant and that this can be attributed to a significant extent to the increased production of extracellular proteases in both mutants. Based on this, we used a mass-based proteomics approach to compare the proteomes of LAC, its isogenic *agr*, *sarA*, and *sarA/agr* mutants, and isogenic derivatives of all four of these strains unable to produce the extracellular proteases aureolysin, SspA, SspB, ScpA, or SplA-F. This allowed us to identify proteins that were present in reduced amounts in *sarA*, and *sarA*/*agr* mutants owing to the increased production of extracellular proteases. A total of 1039 proteins were detected in conditioned media (CM) from overnight cultures of LAC, and protease-mediated degradation was shown to contribute to the reduced abundance of 224 of these (21.6%) in CM from the *sarA* and *sarA/agr* mutants. Among these were specific proteins previously implicated in the pathogenesis and therapeutic recalcitrance of *S. aureus* osteomyelitis. This demonstrates that the ability of *sarA* to limit protease production plays a key role in post-translational remodeling of the *S. aureus* proteome to a degree that can be correlated with reduced virulence in our osteomyelitis model, and that it does so irrespective of the functional status of *agr*. This also suggests that at least some of these 224 proteins may be viable targets for prophylactic or therapeutic intervention.

## 1. Importance

*Staphylococcus aureus* produces a diverse array of virulence factors. This complicates attempts to identify virulence factors that are critical to the pathogenesis of *S. aureus* infections. Mutation of *sarA* limits the virulent *S. aureus* infection including osteomyelitis irrespective of the functional status of *agr*, and an important factor contributing to this attenuation is the increased production of extracellular proteases and the impact of these proteases on its virulence factor repertoire. Identifying proteins whose abundance is limited in *sarA* and *sarA*/*agr* mutants can facilitate efforts to identify virulence factors of particular importance in osteomyelitis and perhaps other forms of *S. aureus* infection. Indeed, the critical role of *sarA* in limiting protease production suggests a mass-based proteomics approach capable of distinguishing between truncated and full-length proteins is the only way to accomplish this task.

## 2. Introduction

*Staphylococcus aureus* is a leading cause of all forms of orthopedic infection including osteomyelitis, septic arthritis, and infections associated with indwelling hardware [1,2,3]. It is becoming increasingly difficult to treat these infections owing to the persistent emergence of strains resistant to key antibiotics. Moreover, these infections are characterized by formation of a biofilm, the presence of which confers a therapeutically relevant level of intrinsic resistance to both conventional antibiotics and host defenses [4,5]. Thus, the treatment of *S. aureus* orthopedic infections requires long-term intensive antibiotic therapy accompanied by surgical debridement to remove infected tissues and indwelling hardware, and even after such intensive medical and surgical intervention, the recurrence rate is unacceptably high [1,2,3].

These observations have rekindled interest in virulence-associated therapies that could be used to overcome this therapeutic recalcitrance [6]. The staphylococcal accessory regulator (*sarA*) and the accessory gene regulator (*agr*) are the two *S. aureus* regulatory loci that have been studied extensively in this regard [6,7,8,9,10]. The *sarA* locus encodes a 15 kDa DNA-binding protein (SarA) that impacts the transcription of many *S. aureus* genes including *agr* [11]. Specifically, SarA is required for maximum expression of *agr*, activation of which modulates the production of multiple *S. aureus* virulence factors in a coordinated fashion [12]. In this regulatory scenario, *agr* is downstream of *sarA*, suggesting that inhibition of *agr* could disrupt the coordinated production of these virulence factors irrespective of the functional status of *sarA*. However, *sarA* also serves regulatory functions that are independent of *agr,* one critical observation in this regard being that mutation of *sarA* results in increased protease production and decreased biofilm formation, while mutation of *agr* has the opposite effect on both phenotypes.

Extracellular proteases produced by *S. aureus* include aureolysin, SspA (V8), SspB, ScpA, and as many as six *spl*-encoded proteases (SplA-F) [13,14]. These proteases collectively serve vital roles on behalf of the bacterium by promoting tissue invasion, nutrient acquisition, and avoidance of host defenses [13,14,15,16,17,18,19]. This suggests that the ability of *S. aureus* to survive in vivo and cause disease would be limited in strains unable to produce proteases. Conversely, strains that produce elevated levels of these proteases would be predicted to exhibit increased virulence. However, a derivative of the methicillin-resistant USA300 strain LAC (Los Angeles County clone) that does not produce any of these 10 extracellular proteases is hypervirulent in murine models of bacteremia and osteomyelitis [13,20], while strains that produce significantly increased amounts of these proteases are attenuated in the same models [20,21,22].

*S. aureus sarA* mutants are the prototype example of the latter in that they produce elevated levels these extracellular proteases, and this has been shown to play a significant role in defining the attenuation of *sarA* mutants in diverse animal models of infection including osteomyelitis [20,22,23,24,25]. Based on this, we used a mass-based proteomics approach to identify proteins that are present in reduced amounts in *sarA* mutants by comparison to their isogenic parent strains owing to protease-mediated degradation [21,26]. Mutation of other *S. aureus* regulatory loci also results in increased protease production, but we confirmed that mutation of *sarA* results in a greater increase in protease production, and a greater decrease in virulence, than mutation of any other *S. aureus* regulatory locus examined to date [23,27,28]. However, these studies were limited to regulatory mutants that exhibit increased protease production, and as noted above this does not include *agr*.

To address this, and to define the impact of *sarA* on protease production in an *agr* mutant, we used our murine osteomyelitis model to directly evaluate the relative virulence of LAC, isogenic *sarA*, *agr*, and *sarA/agr* mutants, and isogenic derivatives of all four strains unable to produce aureolysin, ScpA, SspA, SspB, or SplA-F [20]. The results confirmed that *sarA* and *sarA/agr* mutants are attenuated to a greater extent than the isogenic *agr* mutant, and that this is due to, a statistically significant extent, the increased production of these extracellular proteases. This suggests that *S. aureus* proteins of interest in the specific context of osteomyelitis could be identified and prioritized based on their reduced abundance in both *sarA* and *sarA/agr* mutants by comparison to LAC. Indeed, the reduced virulence of these mutants was correlated with reduced biofilm formation and reduced cytotoxicity for osteoblasts and osteoclasts, and these phenotypes were correlated in turn with the reduced abundance of full-length and presumably functional forms of specific virulence factors implicated in these phenotypes including α-toxin, both components of the Panton–Valentine leukocidin (LukF and LukS), the primary *S. aureus* extracellular nuclease (Nuc1), and protein A (Spa) [20]. However, our studies investigating these correlations were limited by the availability of antibodies appropriate for targeted Western blot analysis. To overcome this limitation, we used a mass-based proteomics approach capable of quantitatively distinguishing between full-length and truncated *S. aureus* proteins [21,26] to assess the global impact of increased protease production on the proteomes of *sarA* and *sarA*/*agr* mutants and to identify *S. aureus* proteins present in reduced amounts in both attenuated mutants owing to protease-mediated degradation.

## 3. Materials and Methods

**Bacterial strains and growth conditions**. Mutants were generated as previously described [20,21,24] and stored at −80 °C in tryptic soy broth (TSB) supplemented with 25% (*v*/*v*) glycerol. Each strain was removed from cold storage and plated on tryptic soy agar (TSA) with antibiotic selection. To prepare conditioned medium (CM), multiple colonies of each strain were used to inoculate TSB without antibiotics. Cultures were grown 16 h and standardized to an optical density (OD_560_) of 10.0. An aliquot was removed and used to verify viable cell density by plating on TSA with and without antibiotics. CM was then prepared for proteome analysis by centrifugation and filter sterilization using 0.22-micron filters.

**Proteome analysis**. Proteins in CM were resolved by SDS-PAGE using 4–12% Bolt Bis-Tris Plus gradient gels (Thermo Fisher Scientific, Waltham, MA, USA). To focus on the abundance of full-length proteins, gels were cut into 12 equal segments and each segment subjected to proteomic analysis as previously described [26]. Briefly, gel slices were destained in 50% methanol (Fisher, Hampton, NH, USA), 50 mM ammonium bicarbonate (Sigma-Aldrich, St. Louis, MO, USA), followed by reduction in 10 mM Tris [2-carboxyethyl]phosphine (Pierce, Woodland Hills, CA, USA) and alkylation in 50 mM iodoacetamide (Sigma-Aldrich). They were then dehydrated in acetonitrile (Fisher), followed by addition of 100 ng porcine sequencing grade modified trypsin (Promega, Madison, WI, USA) in 50 mM ammonium bicarbonate (Sigma-Aldrich) and incubation at 37 °C for 12–16 h. Peptide products were then acidified in 0.1% formic acid (Pierce). Tryptic peptides were separated by reverse phase XSelect CSH C18 2.5 μm resin (Waters, Milford, MA, USA) on an in-line 150 × 0.075 mm column using an UltiMate 3000 RSLCnano system (Thermo). Peptides were eluted using a 60 min gradient from 98:2 to 65:35 buffer A:B ratio (Buffer A = 0.1% formic acid, 0.5% acetonitrile, Buffer B = 0.1% formic acid, 99.9% acetonitrile). Eluted peptides were ionized by electrospray (2.2 kV) followed by MS/MS analysis using higher-energy collisional dissociation (HCD) on an Orbitrap Fusion Tribrid mass spectrometer (Thermo) in the top-speed data-dependent mode. MS data were acquired using the FTMS analyzer in the profile mode at a resolution of 240,000 over a range of 375–1500 *m*/*z*. Following HCD activation, MS/MS data were acquired using the ion trap analyzer in the centroid mode and normal mass range with precursor mass-dependent normalized collision energy between 28.0 and 31.0. Proteins were identified by database search using Mascot (Matrix Science, version 2.6.2, Columbus, OH, USA) against the *USA300 S. aureus* database (2607 entries, Genebank accession JTJK01000002) as previously described [21,26]. Protein probabilities were assigned by the Protein Prophet algorithm [29]. Total spectral counts for each of the 5 biological replicates were exported from Scaffold into Microsoft Excel for further analysis. CM from LAC was used to determine the gel band with the maximum MS2 spectral count for each identified protein. Spectral counts for full-length proteins in other CM were then determined by adding the spectral counts in that gel band with those in the gel bands immediately above and below, thus accounting for minor variations in full-length protein migration through the gel. The spectral counts observed were then averaged for the same gel slices across all biological replicates from all other strains.

**Statistical analysis**. The spectral counts from the gel slice for which the abundance of each protein was highest in CM from LAC was added to the spectral counts for the same protein in the two slices above and below this maximum were used to calculate the fold change and a Student’s *t*-test for significance [26]. The missing value heatmap was generated using R 4.4.1 and the visdat package.

**Western blot analysis**. Western blots were performed using commercially available antibodies (Sigma (St. Louis, MO, USA) and Toxin Technologies (Sarasota, FL, USA), abCAM (Cambridge, UK), and United States Biological (Salem, MA, USA)) as previously described [24].

## 4. Results

**Characterization of mutants**. We first evaluated our results in the context of the mutations that define each of the strains included in these studies. For instance, although SarA is an intracellular protein it was detected at comparable levels in conditioned medium (CM) from overnight (16 h) stationary phase cultures of LAC and its protease-deficient mutant, it was not detected in CM from *sarA* or *sarA*/*agr* mutants or their protease-deficient derivatives (Table 1). AgrA is also an intracellular protein, and it was not detected in CM from LAC or any of its isogenic regulatory mutants irrespective of their ability to produce extracellular proteases. However, delta toxin (δ-toxin), which is encoded within the regulatory RNA associated with *agr* (RNAIII) that is produced under the positive regulatory control of AgrA was detected at comparable levels in LAC and its protease-deficient derivative, but not in CM from the *agr* or *sarA*/*agr* mutants (Table 1). The abundance of δ-toxin was also reduced in CM from the LAC *sarA* mutant by comparison to CM from LAC. This is consistent with studies demonstrating that *sarA* is required for maximum expression of *agr* [11,12]. However, the abundance of δ-toxin was restored in the protease-deficient *sarA* mutant, demonstrating that the increased production of extracellular proteases further limits the abundance of δ-toxin at a post-translational level.

Similarly, the only *S. aureus* proteins encoded outside the *agr* operon and known to be produced under the regulatory control of AgrA are the genes encoding phenol-soluble modulins (PSMs) [30], and all alpha class PSMs (PSMα1, PSMα2, PSMα3, and PSMα4) were absent in CM from the LAC *agr* and *sarA*/*agr* mutants and their protease-deficient derivatives (Table 1). PSMs were present at comparable levels in LAC and its protease-deficient mutant, and absent or only present at very low levels (≤0.6 average spectral counts) in the isogenic *sarA* mutant. However, the abundance of all four alpha class PSMs was increased in CM from the protease-deficient *sarA* mutant. This confirms the importance of *agr* in PSM production, and the importance of *sarA* in limiting proteases to facilitate their accumulation.

The protease-deficient mutants have null mutations in all of the genes encoding aureolysin, ScpA, SspA, SspB, and SplA-F [26], so it would be anticipated that none of these proteases would be detected. We did detect some of these proteases in CM from the protease-deficient *sarA* mutant and, to a lesser extent, the protease-deficient derivative of LAC (Table 1). However, the abundance of these proteases even in CM from the protease-deficient *sarA* mutant was very low (≤1.2 average spectral counts across five biological replicates), and none were detected in the protease-deficient *agr* or *sarA*/*agr* mutants. Our previous phenotypic characterization of these mutants also confirmed that protease activity was limited to the same degree in all four protease mutants [20].

The primary strain in which proteases were detected in a protease mutant was the protease-deficient *sarA* mutant. This is relevant in that every extracellular protease other than SspB was present in an increased amount in CM from the *sarA* mutant by comparison to CM from LAC (Table 1). This suggests that the low levels detected in CM from the protease-deficient *sarA* mutant are background noise. However, the observation that SspB, the abundance of which was decreased in CM from *sar, agr* and *sarA*/*agr* mutants, was detected in CM from the protease-deficient *sarA* mutant contradicts this conclusion. At the same time, we previously demonstrated that spectral counts for full-length SspB are decreased in CM from a *sarA* mutant, but total spectral counts as assessed independently of mass are increased [26]. This is consistent with our previous demonstration that transcription from all four protease-encoding genes and operons is increased in *sarA* mutants [23], and it confirms that mutation of *sarA* results in the increased production of all 10 of the primary extracellular proteases produced by LAC. By comparison to LAC, the abundance of all proteases was reduced in the LAC *agr* mutant (Table 1).

Most proteases were present in CM from the *sarA*/*agr* mutant in amounts comparable to CM from LAC and higher than those observed in the isogenic *agr* mutant (Table 1). However, the abundance of most was reduced in CM from the *sarA*/*agr* mutant by comparison to the isogenic *sarA* mutant. The exception was aureolysin, which was most abundant in CM from the *sarA*/*agr* mutant. This likely accounts for the increase in overall protease activity in CM from the *sarA*/*agr* mutant relative to CM from LAC, while the fact that none of the other proteases were increased likely explains why overall protease activity was decreased in the *sarA*/*agr* mutant by comparison to the *sarA* [20]. Importantly, aureolysin is one of two proteases shown to contribute to the greatest extent to the attenuation of *sarA* mutants in osteomyelitis [24]. The other is staphopain A (ScpA), the abundance of which was increased to a greater extent in the *sarA* mutant than the isogenic *sarA*/*agr* mutant (Table 1). In fact, the abundance of ScpA was not significantly increased in CM from the *sarA*/*agr* mutant by comparison to CM from LAC. Mutation of the genes encoding these same proteases (*aur* and *scpA*) was also shown to replicate the hypervirulent phenotype of a LAC protease null mutant in a murine sepsis model [13].

The fact that the abundance of aureolysin was increased in both *sarA* and *sarA*/*agr* mutants while the abundance of ScpA was only increased in the *sarA* mutant suggests that the increased production of aureolysin may be particularly important in the pathogenesis of osteomyelitis. Indeed, aureolysin-mediated degradation of alpha PSMs was shown to be a primary factor defining the attenuation of a LAC *saePQRS* (*sae*) mutant in osteomyelitis [31]. The reduced abundance of PSMs was also confirmed to contribute to the attenuation of a LAC *sarA* mutant, although whether this could be attributed specifically to degradation by aureolysin was not examined [32].

**Figure 1:**
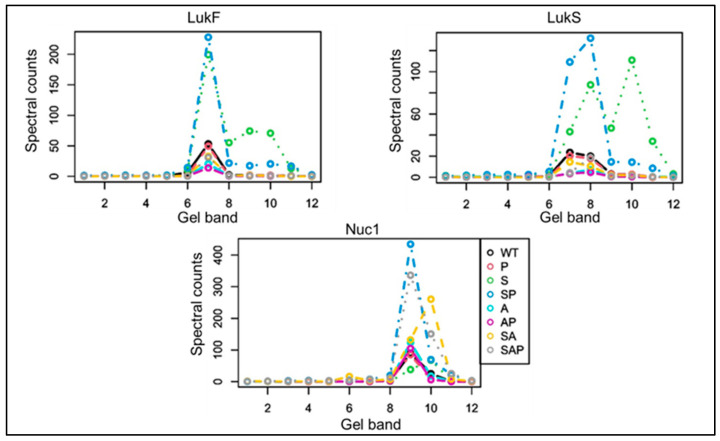
Spectral counts of specific proteins as a function of molecular weight (gel-slice). CM from overnight cultures of LAC, isogenic derivatives with mutations in *sarA* (S), *agr* (A), *sarA*/*agr* (SA), and protease deficient derivatives of all these strains (P, SP, AP, and SAP, respectively) were resolved by SDS-PAGE and each slice subjected to proteomic analysis. Graphs indicate the average spectral count from 5 biological replicates as determined by tandem mass spectrometry plotted for each gel slice.

Our previous Western blots demonstrated that the increased production of extracellular proteases in a LAC *sarA* mutant limits the abundance of LukF, LukS, and Nuc1 in their full-length forms with a corresponding increase in the abundance of a truncated but immunoreactive protein [20]. Using our mass-based proteomics approach, we confirmed the presence of truncated versions of LukF, LukS, and Nuc1 in CM from the *sarA* mutant but not the protease-deficient *sarA* mutant (Figure 1), thus confirming the ability to quantitatively distinguish between full-length and truncated proteins. We also confirmed that these proteins, whether in truncated or full-length form, are present in increased amounts in CM from the *sarA* mutant and its protease-deficient derivative by comparison to CM from LAC (Figure 1), confirming that mutation of *sarA* results in increased production of LukF, LukS, and Nuc1, but that the increased production of proteases limits their abundance in full-length and presumably functional forms.

Additionally,, we previously concluded that LukF is only present in a truncated form in CM from the *sarA* mutant and that this might be a limiting factor in the activity of the bi-component Panton–Valentine leukocidin (PVL) [20]. Here, we confirmed the presence of this truncated product and the fact that it was absent in CM from the protease-deficient *sarA* mutant, but also detected a larger version of LukF that was not apparent by Western blot (Figure 1). This size of this protein was similar to full-length LukF, but it was nevertheless truncated as evidenced by the loss of an antibody-binding site [20]. Thus, this does not contradict the suggestion that the absence of full-length LukF may be a limiting factor in the phenotypic impact of PVL, but it does further illustrate the utility of our mass-based approach. This did not appear to be a concern with LukS and Nuc1 in that both full-length and truncated versions of these proteins were detected by both Western blot and in our proteomics analysis (Figure 1).

**Figure 2:**
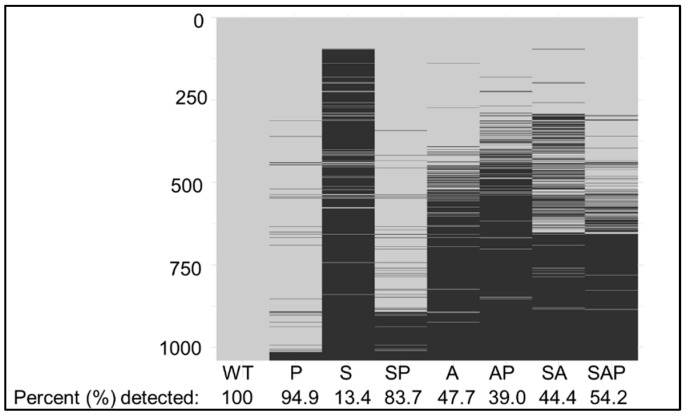
Heatmap reflecting the detection of proteins in LAC and its regulatory mutants as a function of protease production. CM from overnight cultures of LAC, isogenic derivatives with mutations in *sarA* (S), *agr* (A), *sarA*/*agr* (SA), and protease deficient derivatives of all these strains (P, SP, AP, and SAP, respectively). 1039 proteins were detected in CM from LAC. Horizontal black lines in each column indicate proteins that were absent in CM from each mutant, with the percentage of the 1039 proteins detected in CM from LAC that were detected in CM from each mutant indicated.

**Impact of protease production on the proteome of *S. aureus sarA, agr*, and *sarA/agr* mutants**. The results discussed above are consistent with the expected phenotypes of all mutants included in our comparisons. They also validate our mass-based approach including its ability to distinguish between truncated and full-length proteins. Based on this, we extended our analysis in an unbiased, comprehensive, and antibody-independent fashion. We detected 1039 full-length proteins in conditioned media (CM) from stationary phase (16 h) cultures of LAC (Supplementary Tables S1 and S2). Only 139 (13.4%) were detected in CM from the *sarA* mutant, while 870 (83.7%) were detected in the protease-deficient *sarA* mutant (Figure 2). A total of461 full-length proteins (44.4%) were detected in CM from the *sarA*/*agr* mutant, with 563 (54.2%) being detected in CM from the protease-deficient *sarA*/*agr* mutant. The lower number of proteins detected in CM from the *sarA*/*agr* mutant by comparison to the *sarA* mutant is consistent with the observation that protease production is higher in CM from the *sarA* mutant than in CM from the *sarA*/*agr* mutant [20]. Conversely, the fewer proteins detected in CM from the protease-deficient *sarA*/*agr* mutant by comparison to the protease-deficient *sarA* mutant is consistent with the impact of mutating *agr* on protein production. This is further reflected in the reduced number of proteins detected in CM from the *agr* mutant (496), which was not increased in CM from the isogenic protease-deficient *agr* mutant. Indeed, the *agr* mutant was the only regulatory mutant examined in which the number of proteins detected was not increased in the protease-deficient derivative (Figure 2).

**Correlations between protein abundance and virulence**. LAC *sarA* and *sarA*/*agr* mutants are more attenuated in our osteomyelitis model than an isogenic *agr* mutant, and virulence is restored to a significant extent in a protease-deficient derivatives of both mutants [20]. This suggests that proteins that are present in decreased amounts in CM from *sarA* and *sarA*/*agr* mutants owing to protease-mediated degradation would be of interest in the pathogenesis of osteomyelitis. We used a log_2_ fold change >2.0 and *p* value <0.05 to identify these proteins. Because statistical analysis is not possible when considering a protein that was not detected in one of the strains included in the comparison, we also identified proteins that were not detected in CM *sarA* and *sarA*/*agr* mutants but were detected in CM from both of their protease-deficient derivatives (Supplementary Tables S1 and S2).

The ability to identify some proteins of potential interest in CM from the *sarA*/*agr* mutant was limited by the influence of *agr* on protein production. For instance, PSMs contribute to the pathogenesis of osteomyelitis and are present in reduced amounts in CM from *sarA* mutants owing to protease-mediated degradation [32], but the impact of proteases on the abundance of PSMs is not apparent in CM from *sarA*/*agr* mutant because PSMs are not made owing to the *agr* mutation (Table 1). In fact, 543 of the 1039 proteins detected in CM from LAC (52.3%) were not detected in CM from the isogenic *agr* mutant, and virtually all of these were detected at very low levels if at all in CM from the protease-deficient *agr* mutant (Supplementary Table S3). The abundance of an additional 426 proteins was decreased to a significant extent in CM from the *agr* mutant by comparison to CM from LAC (Supplementary Table S4), with the remaining 70 proteins being detected in CM from LAC and its *agr* mutant in comparable amounts (Supplementary Table S5). None of the 426 proteins that were present in decreased amounts in CM from the *agr* mutant were significantly increased in CM from the protease-deficient *agr* mutant. This demonstrates that the reduced abundance of proteins in the *agr* mutant is a function of limited production rather than protease-mediated degradation. Nevertheless, because *sarA* and *sarA*/*agr* mutants are attenuated in our osteomyelitis model owing at least in part to protease-mediated degradation [20], identifying proteins with reduced abundance in *sarA* and *sarA*/*agr* mutants remains important.

**Figure 3:**
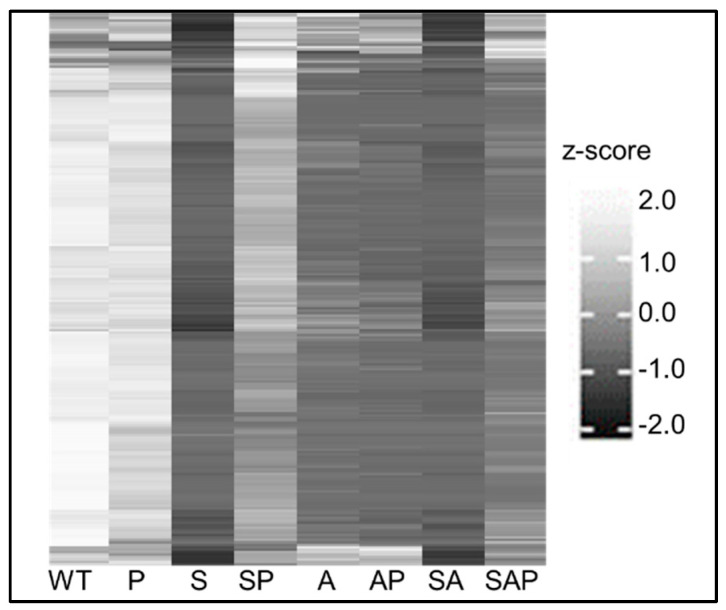
Heatmap reflecting strain dependent variation of prioritized *S. aureus* proteins. Average spectral counts from 5 biological replicates were determined for LAC (WT), isogenic derivatives with mutations in *sarA* (S), *agr* (A), *sarA/agr* (SA), and protease deficient derivatives of all these strains (P, SP, AP, and SAP, respectively). The relative abundance of the 224 proteins that were found to be present in limited amounts in *sarA* and *sarA/agr* mutants owing to protease mediated degradation are shown after transforming data to the Z-scale to reflect the relative differences of each protein to the same protein in all other samples.

A total of 45 proteins were present in a significantly increased amount in CM from the protease-deficient *sarA*/*agr* mutant by comparison to the *sarA*/*agr* mutant itself, with an additional 182 being detected in CM from the protease-deficient *sarA*/*agr* mutant but not in CM from the *sarA*/*agr* mutant (Supplementary Table S6). Thus, the abundance of 227 proteins was limited to some degree by increased protease production in the *sarA*/*agr* mutant (Figure 3). The number of proteins limited by increased protease production in the *sarA* mutant was much greater, but this would be expected given the influence of *agr* on protein production and the observation that protease activity in the *sarA* mutant is higher than in the isogenic *sarA*/*agr* mutant [20].

Of the 227 proteins found to be limited by extracellular proteases in CM from the *sarA*/*agr* mutant, 3 were undetectable in CM from both the *sarA* mutant and its protease-deficient derivative (Supplementary Table S6), thus excluding them from the common pool of proteins present in reduced amounts in both *sarA* and *sarA*/*agr* mutants owing to protease-mediated degradation. Ten of the remaining 224 were present in a significantly increased amount in CM from the protease-deficient *sarA* mutant by comparison to CM from the *sarA* mutant, while 214 were detected in CM from the protease-deficient *sarA* mutant but not in CM from the *sarA* mutant itself (Supplementary Table S6). These results are consistent with the hypothesis that the increased production of extracellular proteases in *sarA* mutants limits the abundance of proteins that are potentially important in the pathogenesis of osteomyelitis, and that it does so irrespective of the functional status of *agr*, suggesting that these 224 proteins may be of particular interest in this regard.

**Impact of eliminating protease production on the proteome of LAC**. While our primary focus was on identifying proteins that are present in reduced amounts owing to increased protease production in *sarA* and *sarA*/*agr* mutants, it was previously reported that eliminating the ability produce extracellular proteases increases the virulence of LAC itself in a murine sepsis model [13]. Since it might be anticipated that this could be attributed to an increase in the abundance of some of the same virulence factors that were limited by protease production in *sarA* and *sarA*/*agr* mutants, we also identified proteins that were present in increased amounts in CM the protease-deficient derivative of LAC by comparison to CM from LAC itself. However, the only two proteins we identified were ArcB and MreC. MreC was among the proteins present in reduced amounts in CM from both the *sarA* and *sarA*/*agr* mutants owing to protease-mediated degradation, but ArcB was not (Supplementary Table S6).

**Figure 4:**
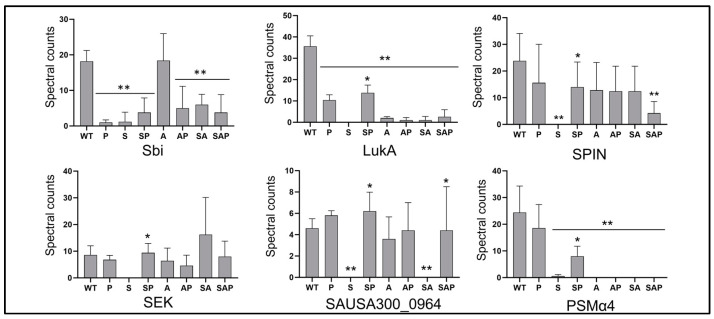
Targeted proteomic comparisons. The abundance of full-length versions of each of the six proteins identified by Gimza et al. [13] was determined using CM from LAC (WT), isogenic derivatives with mutations in *sarA* (S), *agr* (A), *sarA/agr* (SA), and protease deficient derivatives of all of these strains (P, SP, AP, and SAP, respectively). Double asterisks indicate a statistically significant reduction in spectral counts by comparison to CM from LAC as determined by one-way ANOVA. Single asterisks indicate a statistically significant increase in CM from protease deficient derivative of each strain by comparison to its isogenic regulatory mutant as determined by *t*-test.

Additionally, neither ArcB nor MerC were more abundant in CM from the protease-deficient derivative of LAC that was found to be hypervirulent in a murine sepsis model [13]. Moreover, none of the six proteins that were identified in this study (LukA, PSMα4, Sbi, SEK, SPIN, and a putative chitinase encoded by SAUSA300_0964) were identified in our proteomic comparisons of LAC and its protease-deficient derivative (Supplementary Tables S1 and S2). However, the abundance of all six was reduced in CM from the *sarA* mutant, and other than Sbi the abundance of these proteins was increased to a statistically significant extent by eliminating the production of extracellular proteases (Figure 4). The abundance of Sbi, LukA, SAUSA300_0964, and PSMα4 was also reduced in CM from the *sarA*/*agr* mutant, although the reduced abundance of LukA and PSMα4 is likely due to the *agr* mutation (Table 1).

**Figure 5:**
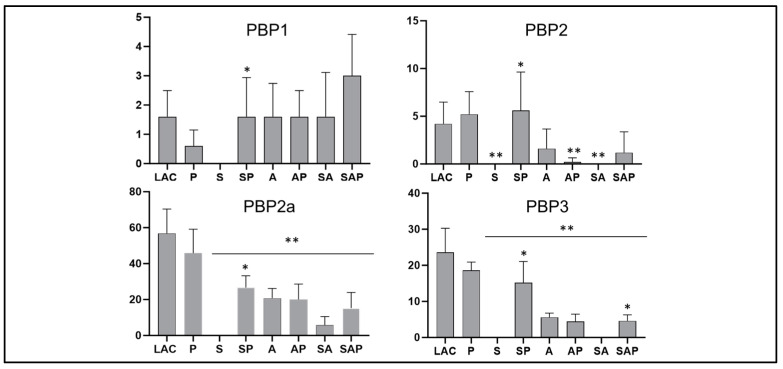
Targeted proteomic analysis of penicillin binding proteins (PBPs). The abundance of full-length PBPs was determined using CM from LAC (WT), its *sarA* (S), *agr* (A), and *sarA/agr* (SA) mutants, and protease deficient derivatives of all 4 strains. Double asterisks indicate a statistically significant reduction in spectral counts as determined by one-way ANOVA comparisons to CM from LAC. Single asterisk indicates a significant increase in the protease-deficient derivative by comparison to its isogenic regulatory mutant as determined by *t*-test.

**Figure 6:**
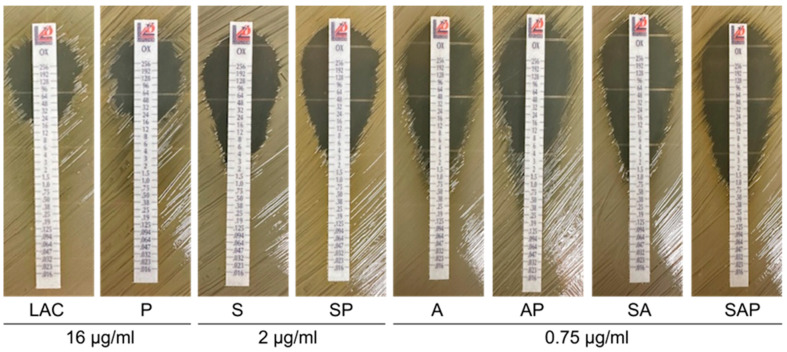
Impact of *sarA*, agr, and extracellular proteases on oxacillin susceptibility. Susceptibility was assessed by E-strip for LAC, isogenic *sarA* (S), *agr* (A), and *sarA/agr* (SA) mutants, and protease-deficient derivatives of all strains (P, SP, AP, and SAP, respectively). The approximate minimum inhibitory concentration shown below the figure was not affected by protease production in any strain.

**Impact of *sarA* and *agr* on oxacillin resistance**. We also found that the penicillin-binding proteins PBP1, PBP2, PBP2A, and PBP3 were detected in CM from LAC but not in CM from its isogenic *sarA* mutant, and that the abundance of all four PBPs was increased in CM from the protease-deficient *sarA* mutant (Figure 5). Mutation of *sarA* increased susceptibility to oxacillin and was increased even more in *sarA*/*agr* and *agr* mutants (Figure 6). However, eliminating the ability to produce extracellular proteases did not impact the susceptibility of any of these regulatory mutants. These results are consistent with a previous report concluding that the impact of *sarA* on oxacillin resistance is a function of decreased transcription of the gene (*mecA*) encoding PBP2A [33]. Irrespective of the mechanism(s) involved, this suggests that targeting *sarA* could have the added benefit of enhancing the ability to overcome the therapeutic recalcitrance of osteomyelitis, particularly when caused by methicillin-resistant strains.

**Figure 7:**
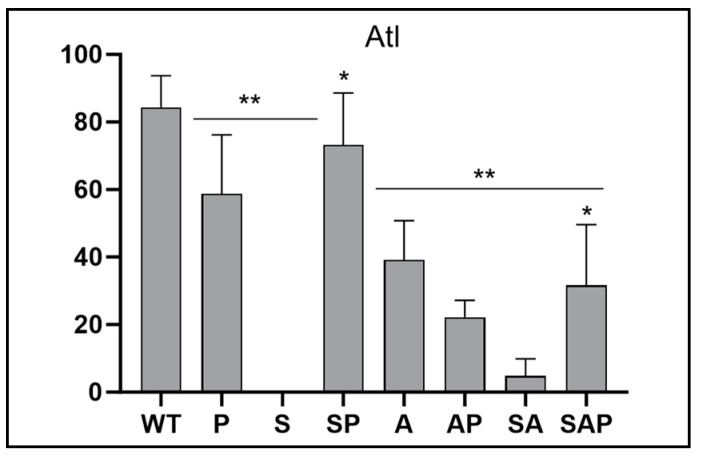
Proteomic comparison for autolysin (Atl). The abundance of full-length Atl was determined using CM from LAC (WT), its sarA (S), agr (A), and *sarA*/*agr* (SA) mutants, and protease-deficient derivatives of all four strains. Double asterisks indicate a statistically significant reduction in spectral counts as determined by one-way ANOVA comparisons to CM from LAC. Single asterisk indicates a significant increase in each protease-deficient strain by comparison to its isogenic regulatory mutant as determined by *t*-test.

**Impact on factors involved in invasion of the osteocyte lacuna-canalicular network (OLCN)**. The reduced abundance of PBP3 may also be relevant in the pathogenesis of osteomyelitis because the absence of PBP3 has been correlated with a decreased ability of *S. aureus* to invade the OLCN [34]. Mutation of the gene encoding the primary *S. aureus* autolysin (*atl*) also limits OLCN invasion, and like PBP3 the abundance of Atl was reduced in CM from *sarA* and *sarA*/*agr* mutants. However, unlike PBP3, the abundance of Atl was restored to a statistically significant extent in CM from the protease-deficient *sarA* and *sarA*/*agr* mutants (Figure 7). Since invasion of the OLCN has been proposed to provide *S. aureus* with a protective niche in bone, this suggests another mechanism by which targeting *sarA* could enhance the ability to overcome the therapeutic recalcitrance of osteomyelitis to conventional antibiotic therapy.

**Figure 8:**
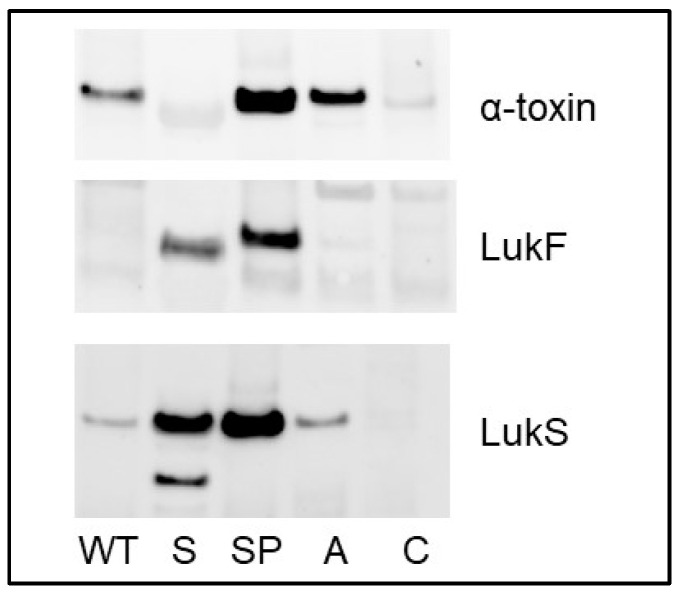
Impact of *atl* on the abundance of *S. aureus* exoproteins. Western blots were done with CM from overnight cultures of LAC and isogenic *sarA* (S), protease deficient *sarA* mutant (SP), an *atl* mutant (A). CM from an *hla* and *lukFS* mutant was included as a control (C).

**Impact on Atl on exoprotein abundance**. It was recently demonstrated that mutation of *atl* results in the increased abundance of extracellular LukAB owing to changes in peptidoglycan cleavage [35]. Atl was essentially undetectable in CM from the *sarA* mutant, so it might be anticipated that the phenotype of the *sarA* mutant would mimic the phenotype of an *atl* mutant. Since eliminating protease production restored the abundance of Atl in the *sarA* mutant, this leaves open the possibility changes in Atl rather than changes in protease production account for the increased abundance of at least some proteins in CM from the protease-deficient *sarA* mutant by comparison to CM from the *sarA* mutant itself. To test this, we used Western blots to examine the abundance of α-toxin, LukF, and LukS in CM from LAC, its *sarA* and protease-deficient *sarA* mutants, and an isogenic *atl* mutant. The results confirmed our previous findings [20] with respect to the decreased abundance of these proteins in their full-length form in CM from the *sarA* mutant and their increased abundance relative to LAC in CM from the protease-deficient *sarA* mutant (Figure 8). They also demonstrated that the abundance of α-toxin and LukS, but not LukF, was increased relative to LAC in CM from the *atl* mutant. However, the abundance of all three exoproteins was still lower in CM from the *atl* mutant than CM from the protease-deficient *sarA* mutant (Figure 8).

## 5. Discussion

*Staphylococcus aureus* produces a diverse array of virulence factors and causes an equally diverse array of infections. This has complicated attempts to identify specific virulence factors that can be studied to gain a better understanding of pathogenesis mechanisms or perhaps even be targeted to prophylactic and/or therapeutic advantage. One alternative is to focus on regulatory elements that modulate the production of multiple *S. aureus* virulence factors, and the two regulatory targets that have been investigated most extensively in this regard are *sarA* and *agr* [6,7,8,9,10]. Although *sarA* is known to function through an agr-dependent pathway [11,12,36,37], the impact of mutating *sarA* and *agr* is often different. One example of specific relevance in this report is their opposite impact on the production of extracellular proteases [38]. Specifically, mutation of *agr* results in decreased protease production, while mutation of *sarA* has the opposite effect.

We have extensively investigated the impact of increased protease production in *S. aureus sarA* mutants and demonstrated that mutation of *sarA* results in a greater increase in protease production, and a greater decrease in biofilm formation, than mutation of any other *S. aureus* regulatory locus identified to date, and that this is true in diverse clinical isolates including both methicillin-sensitive and methicillin-resistant strains [23,27]. These collective studies led us to propose that the ability of *sarA* to repress the production of extracellular proteases is a key mechanism of post-translational regulation in *S. aureus* that prevents these proteases from compromising the *S. aureus* proteome [21,22,23,24,25,26]. However, none of these earlier studies considered the functional status of *agr*, which is an important consideration given that spontaneous *agr* mutants are known to arise in vivo and may in fact promote the transition between acute and chronic forms of *S. aureus* infection including osteomyelitis [39,40,41,42].

To address this, we used our osteomyelitis model to assess the relative virulence of sarA, agr, and sarA/agr mutants generated in the USA300 strain LAC, and the results confirmed that *sarA* and *sarA/agr* mutants are more attenuated in our osteomyelitis model than the isogenic *agr* mutant [20]. This suggests that *sarA* is not only a viable target for therapeutic intervention but is perhaps the preferred target by comparison to other regulatory loci including *agr*. The observation that the reduced virulence of both *sarA* and *sarA/agr* mutants can be attributed to a significant extent to the increased production of extracellular proteases also suggests that a proteomics approach could be used to go beyond the regulatory loci themselves to identify and prioritize specific virulence factors of interest based on a correlation between reduced virulence and the reduced abundance of these virulence factors. In fact, to the extent that our collective studies have also demonstrated that mutation of *sarA* results in the increased production of specific *S. aureus* virulence factors, but limited accumulation of these virulence factors in their full-length and presumably functional forms owing to protease-mediated degradation [20], it suggests that a proteomics approach capable of quantitatively distinguishing between full-length and truncated proteins is the only way to accomplish this important task.

Using this approach, we identified 224 full-length *S. aureus* proteins that are present in reduced amounts in CM from LAC *sarA* and *sarA*/*agr* mutants by comparison to CM from their isogenic protease-deficient derivatives. Many of these proteins were present at very low levels, and in fact most (214) were only identified because they were not detected in CM from *sarA* and *sarA*/*agr* mutants but were detected in CM from their protease-deficient derivatives. The absence of individual proteins in some samples precludes statistical analysis, but we included these among the 224 proteins of potential interest because it is not possible to know how much of a given protein is required to have a phenotypic effect.

The studies we report are an extension of previous studies that used the same mass-based proteomics approach to examine the correlation between protein abundance and virulence in our osteomyelitis model using six isogenic LAC mutants that differed in the functional status of *saeRS* and *sarA* relative to each other [21]. This was based on the demonstration that *saeRS* and *sarA* act synergistically to limit protease production [43] and that mutation of *saeRS* also limits virulence in an osteomyelitis model [21]. In fact, we demonstrated that LAC and its constitutively active *saeRS* variant (*sae^C^*) are more virulent that isogenic *saeRS*, *sarA*, *saeRS*/*sarA*, and *sae*^C^/*sarA* mutants, all of which are attenuated to a comparable degree. Proteomic analysis led to the identification of 114 proteins that were significantly more abundant in CM from both virulent strains by comparison to all 4 attenuated strains. Of these 114 proteins, 25 were among the 224 proteins identified in this study (Supplementary Table S6).

Since mutation of *saeRS* also results in increased protease production, albeit to a lesser degree than mutation of *sarA* [43], it might be anticipated that the overlap between these two datasets would be larger. However, most of the 224 proteins we identified in this study were identified only because they were undetectable in CM from *sarA* and *sarA*/*agr* mutants and detectable in CM from their protease-deficient derivatives, and in our previous study we excluded proteins from further consideration if they were not detected in CM from all 6 strains [21]. Moreover, *sae* also functions in a coordinated fashion with *agr* to regulate the production of *S. aureus* virulence factors in response to different environmental signals [44]. This further illustrates the complex and highly interactive nature of *S. aureus* regulatory circuits and the critical need to maintain an appropriate balance between protein production and protein degradation. This also provides additional evidence supporting the hypothesis that limiting protease production is a key factor in the post-translational remodeling of the *S. aureus* virulence factor repertoire and suggests that the 25 proteins (Supplementary Table S6) that were identified in our collective studies based on correlations with virulence may warrant prioritization even among the 224 identified in this report.

In addition to *sarA* and *saeRS*, mutation of several other *S. aureus* regulatory proteins has been implicated in protease production, and this suggests that additional proteomic comparisons could further enhance the ability to prioritize specific targets for further study. To date, we have examined the impact of 21 *S. aureus* regulatory loci implicated in protease production and biofilm formation. The results demonstrate that mutation of *sarA* results in a greater increase in protease production than mutation of any of these other regulatory loci, but also suggest that *codY*, *sigB*, and *rot* warrant further consideration in this regard [22,25,27]. It is also important to extend such comparisons to other clinically relevant strains. Indeed, the methicillin-sensitive, USA200 strain UAMS-1 is virulent in our osteomyelitis model, and mutation of *sarA* attenuates this virulence owing to increased protease production, but LAC and UAMS-1 differ in significant ways beyond their methicillin-resistance status including their ability to produce important cytolytic including α-toxin and the Panton–Valentine leucocidin [23,24,25,32]. This suggests alternative pathogenesis pathways to the same end, thus making it important to prioritize based not only common proteins impacted by mutation of different regulatory loci but also on common proteins impacted in diverse clinical isolates of *S. aureus*.

Additionally, comparison of datasets obtained independently of each other can be informative, but direct comparisons using the same experimental and statistical methods are clearly preferred, particularly given the sensitivity of current proteomics technology. Indeed, while our primary focus was on identifying proteins that were present in reduced amounts in both *sarA* and *sarA/agr* mutants owing to protease-mediated degradation, we included CM samples from a protease-deficient derivative of LAC itself, and we did not identify any of the six proteins that Gimza et al. [13] found were more abundant in a protease-deficient derivative of LAC by comparison to LAC itself. This could potentially be explained by differences in the specific experimental approaches employed in these two studies, and this accounts for our efforts to fully validate our proteomics approach including its ability to distinguish between truncated and full-length proteins. We also employed a different model to assess virulence, and we did not observe a statistically significant increase in virulence with the protease-deficient derivative of LAC in the specific context of osteomyeltis, but we did demonstrate what we believe are clear trends suggesting increased virulence in the protease-deficient derivative of LAC [20]. Moreover, whether virulence was significantly increased in osteomyelitis vs. sepsis is irrelevant in the context of this discussion since both proteomic comparisons were performed using CM from stationary phase cultures grown in vitro in tryptic soy broth.

There were, however, other differences with respect to sample preparation and analysis that could impact the results. For instance, Gimza et al. [13] standardized cultures based on optical density before precipitating proteins and standardizing samples for comparative analysis based on overall protein content. In contrast, we examined CM after standardization based on optical density without further manipulation based on the logic that standardizing based on protein content might mask important differences owing to the global impact of extracellular proteases. Both studies also used a mass-based approach after resolution by SDS-PAGE, but we used gradient gels and focused on a narrower size range to distinguish between the abundance full-length and truncated proteins [21,24,26]. It is reasonable to suggest that such differences could impact the results, but it is difficult to envision how they can fully explain this discrepancy since we also did not identify any of these six proteins even after removing the size restriction and examining total abundance in any form. Thus, we do not have a definitive explanation for this discrepancy, but this nevertheless further illustrates the need to make direct comparisons between multiple strains and multiple isogenic mutants proven to exhibit significant differences in virulence, and to make these comparisons using consistent methods that have been validated to the greatest extent possible. Given that our proteomics analysis confirmed the identity of all mutants included in our comparisons, and also validated recognized phenotypes beyond the regulatory mutations themselves, we believe the experimental approach we describe is appropriate to address these issues moving forward.

Indeed, while we did not find that any of the six proteins identified by Gimza et al. [13] were more abundant in CM from the protease-deficient derivative of LAC by comparison to CM from LAC itself, we did find that the abundance of four of these six proteins were reduced in CM from the attenuated LAC *sarA* and *sarA*/*agr* mutants. In some cases, the reduced abundance of these proteins in CM from the *sarA/agr* mutant could not be attributed to increased protease production, but this does not detract from the possibility that the reduced abundance of these proteins in CM from *sarA* and *sarA/agr* mutants contributes to their reduced virulence. Indeed, this further illustrates the importance of *agr* in enhancing the production of *S. aureus* virulence factors, and the importance of *sarA* in concomitantly repressing the production of extracellular proteases to maintain a critical balance in the abundance of these virulence factors that is to the best advantage of *S. aureus* in specific in vivo environments including bone.

Identifying critical virulence factors is important because these factors contribute to in vivo survival and ultimately to the damage to the host associated with ongoing infection. To the extent that it compromises the local vasculature, this damage contributes to the therapeutic recalcitrance of *S. aureus* orthopedic infections to conventional antibiotic therapy. However, the limited abundance of these virulence factors is not the only relevant observation in defining this therapeutic recalcitrance. Indeed, penicillin-binding proteins including PBP2A were also identified in this report. This is consistent with previous reports demonstrating that mutation of *sarA* increases β-lactam susceptibility [33]. These reports concluded that the increased β-lactam susceptibility of *sarA* mutants was correlated with reduced *mecA* transcription, and our results demonstrating that the increased susceptibility to oxacillin observed in a *sarA* mutant is not impacted by increased protease production support this conclusion. There is also a report demonstrating that SarA binds the *mecA* promoter [45], further suggesting a protease-independent transcriptional mechanism. However, irrespective of the mechanism involved, it is important to note that mutation of *sarA* increased oxacillin susceptibility to a level comparable to the breakpoint minimum inhibitory concentration (MIC) for a methicillin-resistant strain (≤2.0 µg/mL).

Additionally, PBP3 and the primary *S. aureus* autolysin Atl were also present in low levels in CM from *sarA* and *sarA*/*agr* mutants, and the abundance of both was significantly restored by eliminating protease production. The impact of protease production on the abundance of Atl and PBP3 may be particularly relevant to both the pathogenesis and therapeutic recalcitrance of *S. aureus* osteomyelitis. Specifically, mutation of *atl* and *pbp3* has been shown to limit septic implant loosening and abscess formation in the medullary cavity, while mutation of *pbp3* has been correlated with reduced peri-implant osteolysis, reduced osteoclast activity, reduced invasion of the OLCN, and reduced production of the activator of nuclear factor kappa-B ligand (RANKL), a central modulator of the balance between osteoblast and osteoclast activity in bone remodeling [34,46]. Thus, these observations may be particularly relevant in the specific clinical context of *S. aureus* orthopedic infections.

Finally, one unexpected result we observed is the reduced abundance of SarA in CM from the *agr* and protease-deficient *agr* mutants (Table 1). It has been demonstrated that *sarA* is required for maximum expression of *agr*, but we are unaware of any reports suggesting that *agr* enhances expression of *sarA.* Our studies were performed using a null mutant that does not produce SarA, but different levels of SarA production in different strains has been correlated with relative levels of protease production [47]. Thus, if this were a true regulatory effect with a phenotypic impact, it would be anticipated that the limited production of SarA in the *agr* mutant would result in increased protease production, and this was not the case for any of the 10 extracellular proteases (Table 1). Based on the observation that the abundance of Atl was reduced in CM from the *agr* and protease-deficient *agr* mutants, one plausible explanation is that the reduced abundance of SarA in CM from *agr* mutants reflects changes in the abundance of Atl leading to changes in cell wall turnover and release of intracellular proteins, particularly since our comparisons were performed using CM from stationary phase cultures.

The abundance of SarA was comparable in CM from the *agr* and protease-deficient *agr* mutants, as was the abundance of Atl and SarA (Table 1). Atl abundance was also lower in *sarA* and *sarA*/*agr* mutants by comparison to the isogenic *agr* mutants, and in both mutants it was significantly increased in the isogenic protease-deficient strains. This leaves open the possibility that differences in the abundance of SarA and presumably other proteins in CM from *sarA* and *sarA/agr* mutants by comparison to their protease-deficient derivatives is a function of the abundance of Atl. Indeed, it was recently demonstrated that mutation of *atl* results in the increased abundance of extracellular LukAB owing to changes in peptidoglycan cleavage [34]. Additionally, Atl was essentially undetectable in CM from the *sarA* mutant, so it might be anticipated that the phenotype of the *sarA* mutant would mimic the phenotype of an *atl* mutant. LukA and LukB (SAUSA300_1975 and SAUSA300_1974, respectively) were undetectable in CM from *sarA* and *sarA*/*agr* mutants but readily detectable in CM from their protease-deficient derivatives (Supplementary Table S2). This is consistent with the hypothesis that the abundance of these proteins in CM from *sarA* mutants is limited directly by protease-mediated degradation, but it could also be explained at least to some degree by the limited abundance of Atl in *sarA* and *sarA/agr* mutants.

However, despite the reduced abundance of Atl in CM from the *sarA* and *sarA/agr* mutants by comparison to their protease-deficient derivatives, the abundance of LukF, LukS, and Nuc1 was increased to a comparable degree in both regulatory mutants irrespective of their ability to produce proteases, with the only protease-dependent difference being whether these proteins were present in truncated or full-length and presumably functional forms. Additionally, we confirmed by Western blot that, while mutation of *atl* does result in an increase in the abundance of some exoproteins (e.g., α-toxin and LukS), the abundance of these proteins, as well as LukF, remains below that observed in an isogenic protease-deficient *sarA* mutant. Thus, while we cannot exclude the possibility that changes in the abundance of Atl contribute to differences in the abundance of some of the 224 proteins defined as protease dependent by our comparisons, we do not believe they can account for our cumulative results to an extent that detracts from the conclusion that increased protease production plays a phenotype-defining role in *sarA* and *sarA*/*agr* mutants that includes reduced virulence in the clinical context of osteomyelitis even if differences in the abundance of Atl, which are themselves protease dependent, do in fact contribute to the proteome phenotype of these mutants.

## Figures and Tables

**Table 1:** Spectral count of full-length proteins associated with each mutant. Spectral counts shown are the average of five biological replicates of CM from LAC, isogenic *sarA* (S), *agr* (A), sarA/agr (SA) mutants, and protease deficient derivatives of each strain (P, SP, AP and SAP respectively).

Protein	LAC	P	S	SP	A	AP	SA	SAP
**SarA**	27.6	21.8	0.0	0.0	2.6	2.2	0.0	0.0
**Delta Toxin**	26.6	19.0	8.2	21.4	0.2	0.0	0.0	0.0
**PSMα1**	18.2	19.8	0.4	5.8	0.4	0.4	0.0	0.0
**PSMα2**	14.4	12.6	0.0	3.0	0.0	0.0	0.0	0.0
**PSMα3**	10.2	9.0	0.0	2.6	0.0	0.0	0.0	0.0
**PSMα4**	24.4	18.6	0.6	8.0	0.0	0.0	0.0	0.0
**Aurolysin**	6.4	0.0	14.4	0.0	2.0	0.0	36.2	0.0
**SspA**	19.4	0.2	105.0	1.2	4.6	0.0	19.4	0.0
**SspB**	43.2	0.2	2.0	0.4	5.6	0.0	6.6	0.0
**ScpA**	21.2	0.0	73	0.2	6.2	0.0	29.4	0.0
**SplA**	5.6	0.0	34.6	0.0	0.6	0.0	4.8	0.0
**SplB**	22.4	0.0	107.6	1.0	3.8	0.0	18.4	0.0
**SplC**	6.4	0.0	23.0	0.0	1.2	0.0	3.4	0.0
**SplD**	12.2	0.0	30.8	0.2	3.0	0.0	5.2	0.0
**SplE**	10.2	0.0	30.6	0.0	0.0	0.0	0.6	0.0
**SplF**	10.4	0.0	30.8	0.2	3.0	0.0	5.2	0.0

## Data Availability

The authors confirm that the data supporting the findings of this study are available within this article and its Supplementary Materials.

## Supplementary Materials

The following supporting information can be downloaded at: https://www.mdpi.com/article/10.3390/microorganisms13010181/s1.
